# CAR-T cell therapy in advanced thyroid cancer: from basic to clinical

**DOI:** 10.3389/fimmu.2024.1411300

**Published:** 2024-06-07

**Authors:** Zhenhua Sun, Chaohui Wang, Yuyang Zhao, Qingyi Ling

**Affiliations:** Department of Thyroid and Breast Surgery, Affiliated Hospital of Jiangsu University, Zhenjiang, China

**Keywords:** CAR-T cell therapy, thyroid cancer, biological basis, clinical application, immunotherapy

## Abstract

The majority of patients with thyroid cancer can attain a favorable prognosis with a comprehensive treatment program based on surgical treatment. However, the current treatment options for advanced thyroid cancer are still limited. In recent years, chimeric antigen receptor-modified T-cell (CAR-T) therapy has received widespread attention in the field of oncology treatment. It has achieved remarkable results in the treatment of hematologic tumors. However, due to the constraints of multiple factors, the therapeutic efficacy of CAR-T therapy for solid tumors, including thyroid cancer, has not yet met expectations. This review outlines the fundamental structure and treatment strategies of CAR-T cells, provides an overview of the advancements in both preclinical investigations and clinical trials focusing on targets associated with CAR-T cell therapy in treating thyroid cancer, and discusses the challenges and solutions to CAR-T cell therapy for thyroid cancer. In conclusion, CAR-T cell therapy is a promising therapeutic approach for thyroid cancer, and we hope that our review will provide a timely and updated study of CAR-T cell therapy for thyroid cancer to advance the field.

## 1 Background

Thyroid cancer is the most common malignant tumor of the endocrine system, and its incidence has been increasing significantly year by year; it has become the malignant tumor with the highest incidence rate among women in certain regions (1). The main tissue types are papillary thyroid carcinoma(PTC), follicular thyroid carcinoma (FTC), anaplastic thyroid cancer (ATC), and medullary thyroid cancer (MTC) (2). Due to the expression of sodium iodide symporter (NIS) and thyroid stimulating hormone (TSH)-dependent growth pattern, radioactive iodine 131 (^131^ I) therapy and TSH suppression therapy are effective in most differentiated thyroid cancers. However, treatment options for recurrent/metastatic differentiated thyroid cancer, poorly differentiated/undifferentiated thyroid cancer, and medullary thyroid carcinoma are still insufficient (3).

CAR-T is one of the methods of adoptive cell transfer therapy (ACT); its main principle is to isolate T cells from patients, use genetic engineering technology to insert a chimeric antigen receptor (CAR) into the T cells that can recognize tumor cells and activate the T cells at the same time, and then infuse the expanded CAR-T cells back into the patients and attack the target cells expressing the relevant antigens without relying on the major histocompatibility complex (MHC) (4). As an active medication, CAR-T cell immunotherapy has significantly advanced the treatment of cancer, especially hematologic malignancies. In August 2017, the U.S. Food and Drug Administration (FDA) approved Novartis’ Kymriah, the world’s first CAR-T cell therapy product for the treatment of hematologic malignancies, for the treatment of refractory and relapsed B-cell acute lymphoblastic leukemia (B-ALL) patients (5). The successful application in hematologic malignancies has driven extensive research on CAR-T cell therapy in refractory and relapsed solid malignancies. Several basic studies and clinical trials have demonstrated that CAR-T cell therapy has significantly progressed in thyroid cancer (6). This comprehensive review will present the most recent advancements in CAR-T therapy for thyroid cancer, covering topics such as the biological foundation of CAR-T, ongoing clinical studies, obstacles encountered, and potential solutions to these obstacles.

## 2 Principles of CAR-T therapy and basic structure of CAR-T

T cells depend on the attachment of T cell receptor (TCR) on their surface to antigens presented by MHC molecules on the cell membrane for identifying various cells (7). However, tumor cells evade T cell recognition and killing by reducing or losing MHC expression (8). Conventional ACT approaches, such as tumor-infiltrating lymphocyte (TIL) therapy and T-cell receptor-engineered T-cell (TCR-T) therapy, which recognize only MHC-delivered antigens, may be limited by down-regulation or mutation of MHC molecules of the tumor cells, thus evading immune surveillance, which has certain limitations in the clinic (9, 10). To overcome the limitations of MHC, a promising approach is to modify T cells using CAR to acquire specificity for specific antigenic epitopes, thus enhancing the antigen recognition and activation function of T cells (11). Compared with natural T-cell surface receptors, CAR confers HLA-independent recognition of tumor antigens on T cells, which traditional cellular overlay therapies have unmatched advantages. The U.S. FDA has approved six CAR-T cell therapy medications to treat hematologic cancers (**Table 1**) (18).

**Table 1:** FDA approves CAR-T therapeutics.

Product name	Approval time	Target	Firm	Indications	Complete remission rate	Refs.
Kymriah	2017.8.30	CD19	Novartis	Relapsed or refractory B-group acute lymphoblastic leukemia in adults, Relapsed or refractory large B-cell lymphoma in adults	>90%	(12)
Yescarta	2017.10.18	CD19	Gilead	B-lineage acute lymphoblastic leukemia, Relapsed or refractory large B-cell lymphoma in adults	51%	(13)
Tecartus	2020.7.24	CD19	Gilead	Relapsed or refractory set cell lymphoma in adults	67%	(14)
Breyanzi	2021.2.5	CD19	Bristol-Myers Squibb	Relapsed or refractory large B-cell lymphoma in adults	54%	(15)
Bbecma	2021.3.26	BCMA	Bristol-Myers Squibb	Relapsed or refractory multiple myeloma in adults	28%	(16)
Carvykti	2022.2.28	BCMA	Nanjing Legend Biotechnology	Relapsed or refractory multiple myeloma in adults	78%	(17)

**Figure 1:**
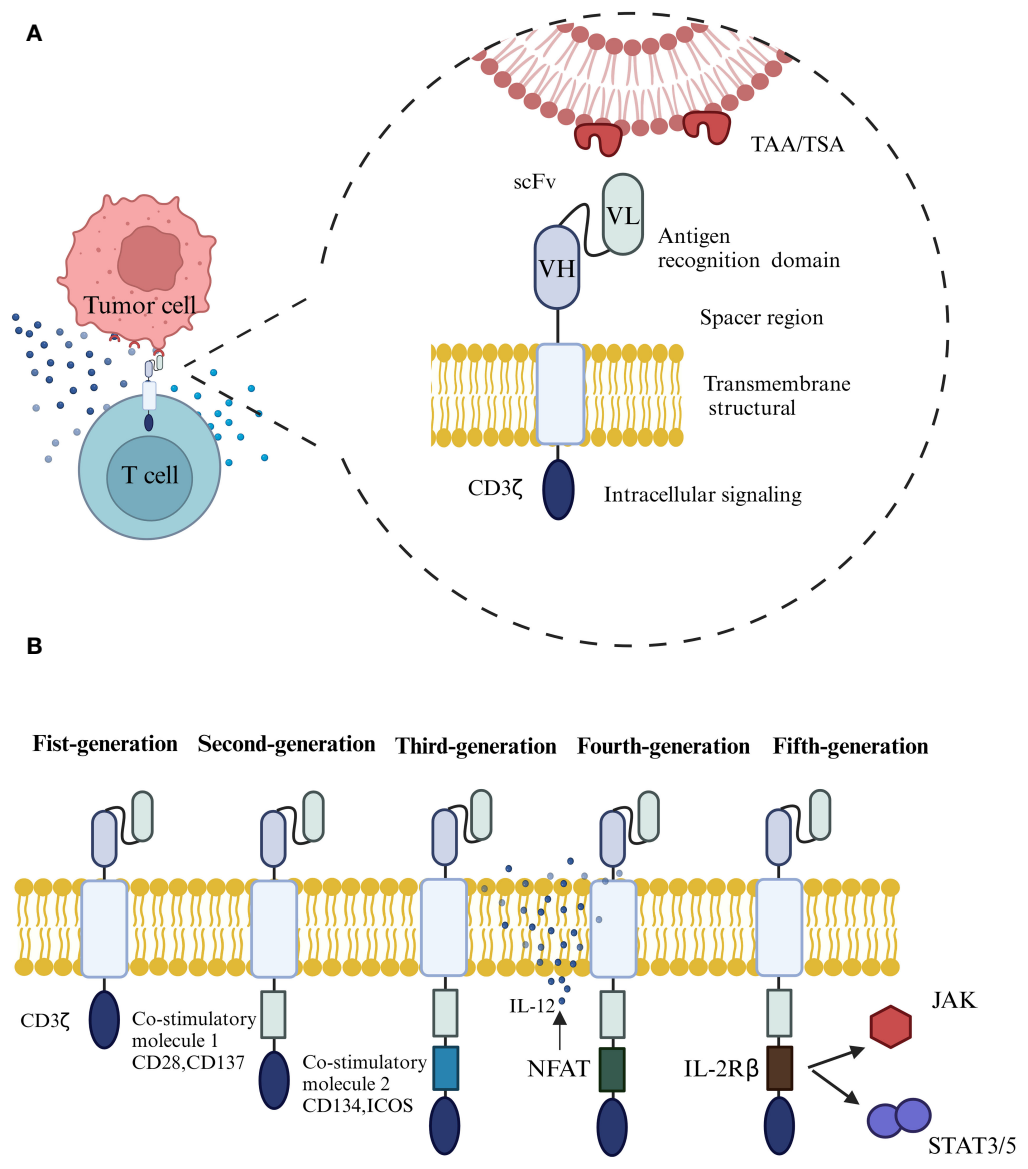
**(A)** The typical structure of CAR consists of four parts, including the extracellular antigen recognition structural domain, the spacer region, the transmembrane structural domain, and the intracellular signaling structural region. **(B)** The development of CAR through the generations, from the first to the fifth generation.

The typical structure of CAR consists of four parts (**Figure 1A**), including the extracellular antigen recognition structural domain, the spacer region, the transmembrane structural domain, and the intracellular signaling structural region (19). The extracellular antigen recognition structural domain is the single chain variable fragment (ScFv), which is the key part of CAR-T to recognize tumor antigen targets and can recognize tumor-associated antigen (TAA) or tumor-specific antigen (TSA). The spacer region, also known as the hinge region, together with ScFv, forms the extracellular structural domain. The transmembrane domain is considered the most important structural feature and generally consists of dimeric membrane proteins that anchor the CAR to the T cell and connect to the intracellular signaling domain (20, 21).

Depending on the design of intracellular signaling structural domains for different purposes, CAR has undergone an evolution from the first to the fifth generation (**Figure 1B**) (22). The extracellular structural domain scFv of first-generation CARs is connected to the intracellular signaling motif CD3ζ through a transmembrane structural domain, which cannot transduce proliferative signals and induce cytokine production due to the absence of co-stimulatory molecule expression, rendering the T-cells unable to increase continuously *in vivo*, The therapeutic effect of tumor-killing is not apparent (23). The second-generation CARs add a co-stimulatory structural domain to its predecessor by adding a co-stimulatory molecule such as CD28, CD137, or an inducible co-stimulatory molecule (ICOS), which allows the T cells to proliferate and release cytokines even in the absence of exogenous co-stimulatory molecules, which can increase the immune memory effect and killing activity (24). Third-generation CARs contain two co-stimulatory structural domains, CD28, CD137, CD134, and ICOS, designed to further enhance signaling capabilities and anti-tumor responses (25, 26). However, it has been shown that the third-generation CART cell-killing activity did not gain significant enhancement, probably because the activation signal generated by a single co-stimulatory molecule in T lymphocytes has reached the threshold, and simply adding co-stimulatory structural domains in quantity will not further enhance the activation effect of CAR on T cells (27). The fourth generation of CAR, also called T cells redirected for universal cytokine-mediated killing (TRUCKs) (28), introduces the activated intra-nuclear factor of T cells (NFAT) based on the previous generation, which can be used to promote the further activation of CAR-T cells in the tumor microenvironment through the production of a series of cytokines, such as interleukin-12 (IL-12), or to add suicide genes or chemokine receptor structures on the structure of CAR-T cells to avoid off-target effects and increase the infiltration of T cells in tumor tissues (29), thus achieving enhanced killing effects on solid tumors. The addition of suicide genes or chemokine receptors to the CAR-T structure can help to avoid off-target effects and increase the infiltration of T-cells in tumor tissues, thus enhancing the killing effect of solid tumors. Based on the second-generation CAR structure, the fifth-generation CAR incorporates co-stimulatory domains that trigger additional signaling pathways, such as the IL-2 receptor β-chain fragment (IL-2Rβ). When the CAR-T cells target tumor antigens, activating the receptor specific to the antigen can activate the downstream JAK-STAT signaling pathway, increasing T-cell proliferation, survival, and anti-tumor activity. While a thorough assessment of the safety and effectiveness of fifth-generation CARs is necessary, their promising potential for advancement has been demonstrated (4, 30).

## 3 CAR-T treatment process

**Figure 2:**
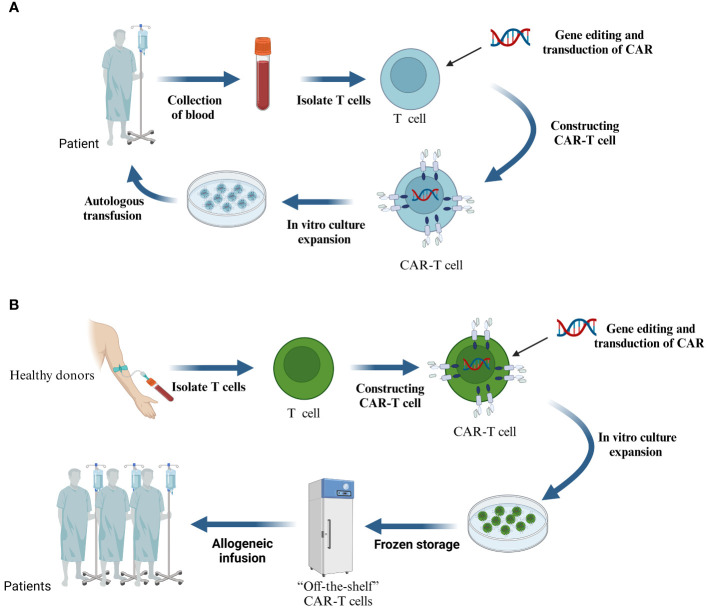
**(A)** The process of treating malignant tumors with CAR-T cells prepared from autologous T cells. **(B)** CAR-T cells are prepared from T cells of allogeneic origin.

CAR-T cell therapy undergoes the following processes (**Figure 2A**): First, blood is extracted from the patient and non-specific T cells are isolated, the isolated T cells are enriched and activated, and then CAR gene transfer is performed using a viral or non-viral vector system that inserts CARs on the surface of the T cells that recognize relevant tumor antigens (31). The reconstructed T cells are cultured and expanded *in vitro*, and finally, the screened and quality-controlled CAR-T cells are infused back into the patient’s body to fight against tumor cells (32). Even though this type of autologous CAR-T cells can successfully evade rejection, the procedure not only requires an extensive preparation period but also comes with a high price tag.”Off-the-shelf” CAR-T cells made from allogeneic T cells are a new alternative (**Figure 2B**) (33, 34). The process begins with the isolation of T lymphocytes from a healthy donor, followed by the transfer and expansion of the CAR gene into the T cells via a viral vector, and finally, quality control, packaging, and frozen storage. Theoretically, allogeneic CAR-T cells are similar to “medicines” and can be used by patients at any time. However, it also faces challenges such as immune rejection and low durability (35).

## 4 Targeting of CAR-T cells for the treatment of thyroid cancer

Tumor antigens can be classified into two groups based on their specificity: TSAs and TAAs (36). Since solid tumors often lack TSA, we have to choose TAA as the target in CAR-T cell design, and solid tumor TAA is expressed on the surface of both tumor cells and normal tissue cells in different degrees. Although this CAR-T target design mode has a certain tumor-killing effect, it is also prone to an “off-target effect”, resulting in serious and long-lasting damage to normal tissues and organs. This “off-target effect” is the main difficulty limiting CAR-T therapy’s clinical application for solid tumors (37). In past research on CAR-T therapy for solid tumors, we tried to avoid tissue-specific antigens in the target screening to avoid the damage of CAR-T cells to normal tissues. Currently, the main clinical treatment strategy for thyroid cancer is to surgically remove the tumor and normal thyroid tissues, followed by oral thyroid hormone replacement therapy (38). Based on this treatment strategy, in selecting CAR-T targets for thyroid cancer, tissue-specific targets expressed on the surfaces of both normal and cancerous thyroid cells can be selected, such as thyroid stimulating hormone receptor (TSHR) and glial-derived neurotrophic factor receptor α4 (GDNFRα4, GFRα4).In addition, non-tissue-specific targets such as carcinoembryonic antigen (CEA), intercellular cell adhesion molecule-1 (ICAM-1), and B7-H3 (CD26), a member of the B7 family of immune checkpoint proteins, are also the main research directions of the current CAR-T cell therapy for thyroid cancer. In the following sections, we will discuss in detail the pre-clinical studies and selected clinical trials of these targets (**Table 2**).

**Table 2:** Ongoing clinical trials of CAR-T therapy for different targets in thyroid cancer.

Clinical Trials ID	CAR-T Strategy	Cancer	Phase	Enrollment	Completion Date	Refs.
NCT 04420754	ICAM−1	Refractory Thyroid Cancer	I	24	2024.12	(39)
NCT 04877613	GFRα4	Recurrent or metastatic MTC	I	18	2039.06	(39)
NCT04119024	IL13Ralpha2	Thyroid Cancer	I	24	2025.10	–
ChiCTR1900025183	TSHR+CD19	Refractory Thyroid Cancer	I	6	2023.12	(40)
ChiCTR1900022607	TSHR	Refractory Thyroid Cancer	I	6	2024.04	–
ChiCTR1900022620	TSHR+CD19	Refractory Thyroid Cancer	I	6	2024.04	(40)

### 4.1 TSHR

TSHR is expressed on the basolateral membrane of thyroid follicular cells and is a member of the transmembrane protein superfamily, G-protein-coupled receptors (41). The development of thyroid cells and their differentiation, as well as the production and release of thyroid hormones, are controlled by TSHR through its interaction with thyrotropin. Research conducted in the past has demonstrated the presence of TSHR in both healthy thyroid tissue and thyroid tumors (42).

Li et al. reported for the first time that TSHR showed good efficacy and safety as a target for CAR-T cell therapy in DTC (40). The researchers first validated the expression of TSHR in tissues and found that TSHR was highly expressed in DTC and low in ATC. Meanwhile, TSHR expression remained high in cervical lymph nodes and refractory thyroid cancer tissues, while it was largely absent in other normal tissues. Given that long-term culture may lead to the transformation of existing differentiated thyroid cancer cell lines into TSHR-deficient types, the researchers constructed TSHR-highly expressing cell lines for *in vitro* experiments. By using pre-existing TSHR antibodies, the researchers screened for scFv capable of targeting TSHR and obtained TSHR CAR-T cells by lentiviral transfection of T cells. After the successful construction, no significant change in the CD4+/CD8+ cell ratio was observed, indicating that the CAR structure was not significantly toxic to the autoimmune system, and the excellent killing effect of CAR-T cells on target cells was evaluated. The research team further established a thyroid cancer model in NSG mice to validate the anti-tumor effect of TSHR CAR-T cells. The outcomes demonstrated that in mice receiving TSHR-CAR-T cell therapy, the tumor volume was considerably decreased. After treatment, the TSHR CAR-T cells increased *in vivo* on days 20 and 34. By monitoring the behavior of the mice and examining their major organs, no significant toxic reactions were observed. Based on this study, a phase I clinical trial entitled “Clinical Study of the Safety and Efficacy of dPD-1 (TSHR+CD19) CART Cells in the Treatment of Relapsed Refractory Thyroid Cancer” has been conducted, which has been completed, and the results have not yet been reported.

2022 Ding et al. reported a clinical case of TSHR+CD19 dual-targeted CAR-T cells to treat one case of recurrent refractory thyroid cancer (43). A female patient with poorly differentiated thyroid cancer received a TSHR+CD19 CAR-T cell infusion. Imaging on day 30 after infusion suggested no tumor progression and partial remission occurred on day 90. Dynamic monitoring of the patient’s *in vivo* CAR-T cell proliferation revealed that the number of CAR-T cells continued to increase after infusion, reaching a peak on day six and remaining detectable for three months. The patient died of a lung infection on day 141 post-infusion, but the efficacy of thyroid cancer treatment was still evaluated as a partial remission (PR) before the patient’s death. Overall, TSHR+CD19CAR-T cells expanded well *in vivo* in humans and have promising efficacy with aggressive control of adverse effects.

### 4.2 ICAM−1

ICAM-1, a cell surface glycoprotein and adhesion receptor, belongs to the immunoglobulin superfamily and is found on various cells. It plays a crucial role in controlling the migration of white blood cells from the bloodstream to inflammatory areas. It involves essential physiological processes such as cell signaling and activation, immune response, and inflammatory response. Some studies have shown that ICAM-1 expression is increased in a variety of tumors, including thyroid, pancreatic, and breast cancers, and is involved in biological processes such as tumor invasion and metastasis (44, 45). Previous studies have shown that CAR-T cells constructed against overexpressed ICAM-1 have excellent anti-tumor efficacy and safety and thus are expected to be a target for tumor CAR-T therapy (46–48).

In 2017, Min et al. reported the first study of CAR-T therapy for thyroid cancer utilizing ICAM-1 as a target (49). *In vitro* experiments, ICAM-1 CAR-T cells demonstrated efficient killing effects on ICAM-1-expressing papillary thyroid cancer and undifferentiated cancer cell lines. As verified by the metastatic tumor animal model constructed in NSG mice, ICAM-1, CAR-T significantly inhibited the growth of tumor cells and exhibited durable anti-tumor activity, prolonging the survival of mice. In addition, the killing effect of autologous ICAM-1 CAR-T on thyroid malignant tumor cells *in vivo* and *in vitro* was verified. Studies in cytotoxicity revealed that ICAM-1 CAR-T had no significant killing effect on non-tumor cells and that endogenously expressed ICAM-1 had no significant adverse effect on the autoimmune system. After infusing ICAM-1 CAR-T cells into immunodeficient mice, no significant damage was observed to essential tissues such as lungs and livers. In summary, CAR-T cell therapy for refractory and recurrent thyroid cancer has significant potential with ICAM-1 as a target.

In follow-up studies, Min and his team optimized the production process of ICAM-1 CAR-T cells, improved the cell structure, and conducted an in-depth evaluation of their efficacy and safety. This ultimately provided sufficient preparation for developing phase I clinical trials (50). In 2020, AffyImmune Therapeutics, Inc. of the U.S. is conducting a Phase I clinical trial of ICAM-1 CAR-T cells for the treatment of patients with relapsed/refractory poorly-differentiated thyroid cancer and patients with BRAF wild-type undifferentiated thyroid to evaluate the safety and tolerability of its product AIC100, the trial is expected to last until June 2024. Reports from this preclinical trial indicated that AIC100 amplified well in ATC and PTC patients, demonstrated an excellent safety profile, and effectively killed tumors (51, 52).

### 4.3 GFRα4

GFRα4 is a member of the glial-derived neurotrophic factor (GDNF) family of receptors alpha expressed in normal and malignant medullary thyroid cells in humans. When GFRα4 binds specifically to the GDNF family ligand persephin (PSPN), it induces phosphorylation of RET proteins, activating downstream signaling pathways and exerting its physiological effects (53). Given the restricted expression profile of GFRα4 in MTC (54), it may be a potential specific and relevant antigenic target for CAR-T treatment of MTC.

A study by Bhoj et al. reported that GFRα4 expression in humans is restricted to parafollicular thyroid cells and was detected in the tumors of all MTC patients tested (55). Due to the lack of specific monoclonal antibodies, the investigators performed a structural screen of scFv against GFRα4 isoforms and successfully obtained GFRα4 CAR-T cells by lentiviral transfection of T cells. *In vitro* experiments verified the specificity of the CAR structure binding to GFRα4 and observed that cells expressing the CAR structure could be activated by MTC cells. Further studies showed that the GFRα4 CAR-T had a killing effect on MTC cell lines and leukemia cell lines with high expression of GFRα4 and secreted IL-2 and interferon γ (IFN-γ). In *in vivo* experiments, the researchers found that GFRα4 CAR-T cells could effectively inhibit tumor growth in the constructed MTC cell line NSG mouse xenograft animal model. However, GFRα4 CAR-T cells expanded to a greater extent, possibly related to off-target effects. In terms of *in vivo* toxicity, GFRα4 CAR-T cells exhibited toxicity against the skin of NSG mice, but no significant toxicity manifestation was observed in other tissues and organs. Further studies showed that, unlike mouse epithelial cells, human epithelial cells, as well as other cells except for MTC, could not activate GFRα4 CAR-T cells. The skin toxicity may result from the off-target response of CAR to unknown antigens in mice but not human keratinocytes. Subsequent studies humanized scFv and validated its specific binding to GFRα4 as well as its efficacy *in vitro* and *in vivo* and skin toxicity (56). In 2021, the University of Pennsylvania initiated a Phase I clinical trial of single-stranded scFv CAR-T cells targeting GFRα4 for the treatment of recurrent or metastatic MTC to evaluate the safety and feasibility of the product and to find the maximum safe dose, with a trial cut-off date expected to be June 2039 (39).

### 4.4 CEA

CEA is a tumor-associated antigen, initially thought to be a proteoglycan complex present in colon cancer and normal embryonic intestines, and later shown to be widely present in digestive tumors of endodermal origin. Its expression is positively correlated with the malignancy of the cancer. It is also found in trace amounts in the blood of ordinary people, so CAR-T therapy targeting CEA needs to consider its possible adverse effects (57). CAR-T cell therapy targeting CEA has demonstrated significant efficacy in early studies and clinical trials against metastatic liver cancer, colorectal cancer, and other tumors (58). Erickson et al. (59)reported the design and synthesis of a CAR-T cell targeting CEA for targeted therapy against MTC. The results of *in vitro* experiments clarified the binding ability of CEA CAR-T cells to their target. They confirmed that these cells had a significant killing effect on CEA-expressing positive MTC cells but not CEA-negative ones. In addition, high levels of IFN-γ, tumor necrosis factor (TNF), and IL-2 expression also demonstrated the practical killing effect of CEA CAR-T cells on target cells. The killing ability of CEA CAR-T cells against CEA-expressing positive MTC cells was further validated by an animal model of MTC established in NSG mice. However, its safety still needs to be further evaluated. No relevant clinical trials for CEA CAR-T cell therapy against MTC have been registered.

### 4.5 B7-H3

B7-H3, an immunological checkpoint protein belonging to the B7 family, is expressed significantly in a range of primary malignancies and exhibits restricted expression in normal tissues (60). As a co-stimulatory molecule, B7-H3 provides a second signal in T-cell proliferation and activation. However, its effects on T-cells appear to have multiple effects. Recent research indicates that B7-H3 plays a key role in immune evasion, tumor spread, angiogenesis, and resistance to treatment in cancers. This suggests that targeting B7-H3 could be a promising strategy for tumor immunotherapy, particularly in MTC (61). The utilization of the pan-cancer antigen B7-H3 as a target for CAR-T cells has demonstrated effective preclinical activity in the treatment of a variety of malignant tumors. Duan et al. designed a CAR-T cell targeting B7-H3 (62), which tandemly linked fragment of antigen binding (Fab) and natural TCR intracellular signaling structural domains to form a novel Fab CAR, which recognizes MHC-independent tumor antigens and mimics the natural activation process of endogenous TCR. This design effectively addresses the problem of premature T-cell depletion. The study findings indicated that Fab CAR-T cells could efficiently target cancerous cells of the human follicular thyroid and induce cytotoxic effects. Unfortunately, the experiment was not evaluated for safety in animal studies. Further animal experiments are needed to evaluate the safety of Fab CAR-T cells, which will help to comprehensively evaluate their efficacy and potential application prospects.

### 4.6 Other potential targets

In addition to the targets mentioned above, no additional studies on CAR-T cell therapy for thyroid cancer have been reported. Based on the principles of CAR construction (63), we speculate that pan-tumor markers such as NIS, thyroglobulin(Tg), thyroid peroxidase(TPO), proto-oncogene tyrosine-protein kinase receptors(RET), calcitonin(CT), protein kinase B(PKB), telomerase reverse transcriptase (TERT), and the kinetochore element Ndc80 could be potential targets for thyroid cancer immunotherapy (59, 64). However, further preliminary studies and clinical trials need to validate these speculations.

## 5 Challenges and strategies for CAR-T cell therapy for thyroid cancer

Despite demonstrating potential in both preclinical research and clinical trials as a treatment for thyroid cancer, the application of CAR-T cell therapy continues to encounter numerous obstacles. These challenges mainly include the lack of specific antigens and tumor heterogeneity, deficient infiltration of CAR-T cells within tumors, immunosuppression of the tumor microenvironment (TME), and toxic side effects of CAR-T cells (65, 66). To address these issues, researchers have proposed strategies to promote further CAR-T cell therapy’s use in treating solid tumors.

### 5.1 Specific antigen deficiency and tumor heterogeneity

A key factor in immune cell therapy’s remarkable success in treating hematologic tumors is its ability to target well-defined, uniformly expressed, and tumor cell-specific antigens. In contrast, in solid tumor therapy, fewer TSAs are present, mostly TAA, and these antigens are not only expressed in tumor cells but can also be found in normal tissues, thus increasing the risk of off-targeting (36). In addition, the heterogeneity of expression intensity and distribution exhibited by solid tumor antigens in the immune microenvironment makes it challenging to ensure efficacy even when ideal targets are identified (67, 68). In addition to searching for and developing new targets with higher specificity, the lack of specific antigens can be addressed by modifying CAR-T cells to improve their ability to recognize tumor antigens. Wendell A Lim’s team implanted the synNotch system in CAR-T cells. Under the regulation of synNotch, CARs recognizing the relevant TAA will only be expressed on T cells migrating into tumors and will not attack cells in normal tissues, improving the specificity of CAR-T recognition of antigens (66). In addition, various traditional epigenetic modulators have been shown to modulate antigen density, thereby increasing the sensitivity of CAR-T to antigen recognition. For example, Decitabine, a DNA methylation transferase inhibitor, can enhance the specific recognition and killing effect of MUC1 CAR-T by upregulating the expression of MUC1 antigen on pancreatic cancer cells (69). Various strategies for modifying CARs have been developed to target antigenic heterogeneity in solid tumors, one of which is the simultaneous targeting of multiple tumor antigens to provide a higher level of antigen recognition ability for infused immune cells, such as dual-targeted tandem CARs recognizing EpCAM and ICAM-1 (70), and triple-targeted CARs targeting three antigens, namely, HER2, IL-13Rα2, and EphA2, at the same time (71). Secondly, EGFRvIII -CAR-T cells that secrete bispecific T-cell engager (BiTE) targeting EGFR can circumvent toxicity and improve anti-tumor efficacy against heterogeneous glioblastoma (72). These approaches have shown better efficacy in preclinical models.

### 5.2 Inefficient transit of CAR-T cells in thyroid cancer therapy

Infiltration of immune cells into the tumor site is a prerequisite for their effector function. The highly abnormal vascular and stromal structures in the microenvironment of solid tumors are thought to be the main factors hindering the infiltration of CAR-T cells. Tumor vasculature usually exhibits an irregular shape with varying degrees of collapse, whereas the tumor stroma is much denser. These two constitute a physical barrier resulting in brutal penetration of CAR-T cells (73). In addition, some solid tumors inhibit the secretion of specific chemokines. The interaction of chemokines with their receptors promotes the migration of T cells to the tumor microenvironment (74). Concurrently, CAR-T cells also lack pertinent surface receptors that correspond to the chemokines released by solid tumors, resulting in inadequate CAR-T cell infiltration at tumor sites. Recent studies targeting chemokines have opened up new possibilities for CAR-T cell infiltration of solid tumors. Overexpression of chemokines by pretreatment of tumors with chemotherapeutic agents or modification of CAR-T cells can increase endogenous immune cell infiltration at the tumor site (75). Similarly, modification of the chemokine receptors CXCR1/CXCR2/CXCR5 to the surface of CAR-T cells can increase cellular transport within the tumor and anti-tumor efficacy (76–78). Modification of CAR-T cells by strategies targeting the tumor stroma, such as using ECM-degrading agents such as acetyl heparinase or fibroblast activating proteins (79, 80), enhances their ability to penetrate physical barriers and thus increases aggregation in solid tumors. In addition, multidisciplinary cross-fertilization opens up new ideas to break the dilemma of insufficient immune cell infiltration in the tumor microenvironment, such as intratumoral injection (81), implantable scaffolds (82), and special biomaterials delivery (83), etc., which can enable the infused immune cells to be delivered to the tumor site more efficiently and effectively and target the tumor as well as reduce the off-target effect.

### 5.3 Immunosuppression of the tumor microenvironment

TME is a microenvironment that promotes the growth of cancer cells and consists of tumor cells, inflammatory cells, fibroblasts, mesenchymal tissues, and various cytokines. CAR-T cells can reach their destination directly after entering the blood circulation for hematologic tumors (74). However, for solid tumors, the tumor microenvironment not only has a physical barrier to inhibit T-cell infiltration but also contains a variety of immunosuppressive cells and immune checkpoints (84). This environment predisposes CAR-T cells to poor migration and persistence, impaired cellular function, and cellular exhaustion, thus failing to kill tumor cells effectively (85). Low-dose chemotherapy, a primary pretreatment strategy in present-day clinical CAR-T therapy, can potentially alter the tumor’s immune microenvironment. This modification can heighten CAR-T effectiveness through the removal of immunosuppressive cells (86, 87). In addition, studies have shown that CAR-T efficacy can be improved by modifying CARs to release immunostimulatory cytokines (88) or by interfering with immunosuppressive cytokines and inhibitory signaling pathways to counteract TME-induced immunosuppression (89). Immune checkpoints present an alternative solution for assisting CAR-T cells in overcoming the immunosuppressive conditions within solid tumors (90). Combining programmed death 1(PD-1) blockers with CAR-T cells that target ICAM1 enhances the efficacy of eliminating ICAM1-expressing thyroid tumor cells compared to using only CAR-T therapy (91). In addition to combining immune checkpoint inhibitors, it is also possible to construct CAR with anti-programmed cell death-Ligand 1 (PD-L1) scFv sequences (92), which are CAR-T cells that kill tumor cells while also blocking the PD-1/PD-L1 signaling pathway to release the immunosuppressive state.

### 5.4 CAR-T cell therapy-related toxicity

While CAR-T cell therapy has demonstrated revolutionary potential in cancer treatment, its elevated toxicity rates have hindered its widespread adoption as a primary clinical intervention, with the main types of toxicity being targeted tumor toxicity, non-tumor-targeted toxicity, off-target toxicity, and neurotoxicity (93). In addition, the FDA’s latest announcement states that all approved CAR-T cell therapy products targeting BCMA or CD19 carry a malignant risk of secondary T-lymphoblastic neoplasms (94). However, this risk has not been reported in solid tumors. Several approaches have been developed in recent years to address the issue of toxicity, including modification of CAR-T cell structure, modification of CAR-transduced T cells, implementation of a CAR “off switch,” or introduction of a suicide gene. By modifying the modular structure of CAR-T cells, it is possible to reduce their affinity for antigens, thus avoiding targeting normal tissues, and at the same time regulating the secretion of cytokines and reducing the immunogenicity of CAR, to achieve the purpose of controlling the toxicity of CAR-T cell therapy (95–97). Mitigation of CAR-T cell toxicity can also be achieved by implementing an “off switch” or introducing a suicide gene that selectively reduces genetically modified cells upon the occurrence of an adverse event by treatment with a secondary inducer, e.g., by inducing caspase 9 (iCasp9) and CD20 as a safety switch to remove CAR-T cells by rituximab treatment. Shikinumab treatment removes CAR-T cells, thereby reducing their toxicity (98). Another promising approach involves using the tyrosine kinase inhibitor Dasatinib to reversibly activate T cells by inhibiting proximal TCR signaling kinases (99). T-cell activation is ensured while reducing toxicity.

## 6 Conclusion and outlook

Currently, immunotherapy is not yet used as a conventional treatment for thyroid cancer. Still, for those patients who are in advanced stages of the disease and for whom other treatments have not been effective, novel therapeutic regimens, represented by CAR-T, may offer a new avenue for them and clinical practitioners. The purpose of this article is to review the development of CAR-T cell therapy for thyroid cancer from basic research to clinical trials. In preclinical studies, CAR-T cells constructed with antigens such as TSHR, ICAM-1, GFRα4, B7-H3, and CEA demonstrated anti-tumor effects. Still, only three antigens have entered clinical trials, and some relevant results have not yet been published. Further, fundamental studies and clinical trials are required to refine the CAR architecture for improved T cell activation, recognition specificity, anti-tumor activity, and safety control to achieve a reliable cure for solid tumors such as thyroid cancer. Finding optimal signaling and co-stimulation regions to enhance CAR-T therapy efficacy is crucial. In addition, there is a need to overcome the immunosuppressive tumor microenvironment with additional modifications to CAR-modified T cells. Establish standard clinical protocols, including patient pretreatment, cytokine support, and other potential combination therapies (100, 101). Through these efforts, we expect to make reliable advancements in CAR-T cell therapy in thyroid cancer and provide better treatment options for patients.

## Author contributions

ZS: Writing – original draft, Writing – review & editing. CW: Writing – original draft, Writing – review & editing. YZ: Writing – original draft. QL: Writing – original draft.
